# *Pneumocystis jirovecii* Pneumonia in a HIV-Infected Patient with a CD4 Count Greater Than 400 Cells/*μ*L and Atovaquone Prophylaxis

**DOI:** 10.1155/2020/8532780

**Published:** 2020-07-12

**Authors:** Abigayle Sullivan, Theresa Lanham, Ronald Krol, Shilla Zachariah

**Affiliations:** ^1^Department of Internal Medicine, Reading Hospital-Tower Health System, West Reading, PA, USA; ^2^Department of Pulmonary and Critical Care, Reading Hospital-Tower Health System, West Reading, PA, USA

## Abstract

We describe a rare case of *Pneumocystis jirovecii* pneumonia (PCP) in a heterosexual man with a pertinent medical history of well-controlled human immunodeficiency virus (HIV) on highly active antiretroviral therapy (HAART) and PCP prophylaxis with atovaquone. The patient presented with recurrent shortness of breath, worsening malaise, and fever, following treatment for hypersensitivity pneumonitis one month prior, including a twenty-four-day course of 40 milligrams daily glucocorticoid with taper. However, transbronchial biopsies, lavage, and cytology from prior admission were inconclusive. The patient refused video-assisted thoracic surgery (VATS) at that time. Upon readmission, bronchoscopy with right VATS and lung biopsy were performed. Grocott's methenamine silver stain of right lung biopsy was positive for *Pneumocystis jirovecii*. This case is a rare example of PCP in a patient with a normal CD4 count (>487 cells/*μ*L) and a low viral load (<20 copies/mL) despite PCP prophylactic antibiotics in the setting of recent iatrogenic immunosuppression.

## 1. Background

*Pneumocystis jirovecii* pneumonia is one of the most common pulmonary pathogens and a leading cause of opportunistic infection in patients infected with HIV. Immunosuppressed individuals, including those infected with HIV with a low CD4 count, solid organ transplant recipients, and those prescribed with immunosuppressive medications, are at a substantial increased risk of developing PCP. The incidence of PCP has substantially decreased after HAART and antibiotic PCP prophylaxis. Current guidelines recommend prophylactic treatment for patients with a CD4 count less than 200, although some studies have shown no incidence of infection of prophylaxis with a CD4 count of 101–200 cells/microliters and undetectable HIV ribonucleic acid (RNA). However, there have been few selected cases describing PCP in immunocompetent patients.

## 2. Case Report

**Figure 1:**
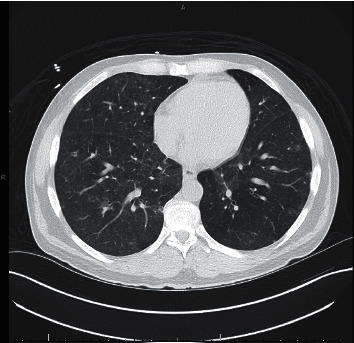
Computerized axial tomography chest illustrating bilateral ground glass opacities from first admission.

**Figure 2:**
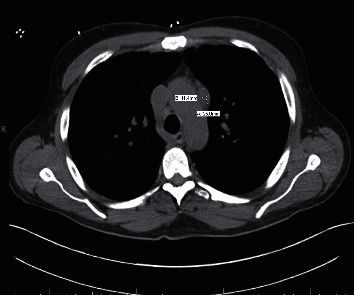
Computerized axial tomography chest demonstrating intrathoracic lymphadenopathy from first admission. One lymph node measured to be 11.4 mm by 25 mm.

**Figure 3:**
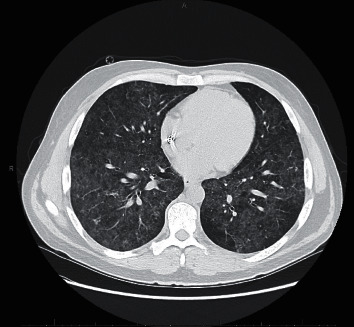
Computerized axial tomography chest showing persistent bilateral extensive ground glass opacities as well as improved mediastinal lymphadenopathy.

We present a 39-year-old heterosexual man with a pertinent past medical history of well-controlled HIV on HAART, including dolutegravir, lamivudine, and abacavir, and atovaquone prophylaxis and end-stage renal disease on hemodialysis. Atovaquone prophylaxis, 750 mg PO every 12 hours, was initiated 5 months prior when the patient was first diagnosed with HIV with CD4 count 81 cells/*μ*L and acute renal failure. The patient first presented to the hospital for evaluation of shortness of breath. During this admission, computerized axial tomography (CT) chest demonstrated interstitial lung disease with ground glass opacities and intrathoracic lymphadenopathy (Figures 1 and 2). The patient underwent bronchoscopy, which was suboptimal due to technical difficulties. Transbronchial biopsies, bronchoalveolar lavage, and cytology were inconclusive. Furthermore, acid-fast bacilli (AFB) cultures were negative. Unfortunately, the patient declined VATS. In addition, the patient had significant peripheral eosinophilia 11.8% (ref.: 0.0–6.0) and occasional urine eosinophils (ref.: negative). At this time, *Pneumocystis jirovecii* pneumonia was not suspected due to normal CD4 count 487 cells/*μ*L with atovaquone prophylaxis and HIV RNA <20 copies/mL. Other diagnosis considerations included iatrogenic-induced subacute hypersensitivity pneumonitis and sarcoidosis. Abacavir was switched to rilpivirine due to suspicion of hypersensitivity pneumonitis, and the patient was discharged on 40 milligrams prednisone daily, and atovaquone prophylaxis was continued due to prednisone therapy. Following these treatments, there was both a radiographic and clinic response. The patient received a twenty-four-day course of prednisone. Follow-up CT chest demonstrated persistent, extensive bilateral ground glass opacities and improved mediastinal lymphadenopathy (Figure 3).

The patient presented back to the hospital ten days after discontinuation of prednisone for evaluation of worsening shortness of breath, malaise, and fever. On presentation, the patient was hypoxic (oxygen saturation 88% on room air), febrile (38.8°C), tachycardic (heart rate low 100 s), and tachypneic (29 breaths per minute). Physical exam was remarkable for increased work of breathing and diffuse bilateral rales.

**Figure 4:**
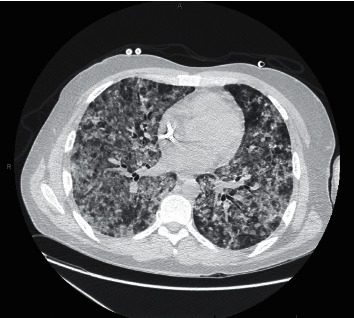
Computerized axial tomography chest highlighting significantly worsened bilateral ground glass opacities and dense airspace consolidations.

Repeat CT scan showed substantially worsened diffuse bilateral ground glass and more dense airspace consolidations (Figure 4). A bronchoscopy with right VATS and lung biopsy was performed. Cytology was negative for malignancy, and AFB stain was negative. Grocott's methenamine silver stain of pathology from right upper, middle, and lower lobe resulted positive for *Pneumocystis jirovecii*.

The patient's atovaquone for PCP prophylaxis was discontinued, and renally dosed trimethoprim-sulfamethoxazole 5 mg/kg/day (trimethoprim component) IV was initiated. The patient was discharged on 2 tablets of oral trimethoprim-sulfamethoxazole (160–800 mg tablet) daily for a total course of twenty-one days, in addition to a short course of prednisone. The patient experienced significant relief of shortness of breath following completion of trimethoprim-sulfamethazine course and prednisone taper. PCP prophylaxis was changed to trimethoprim-sulfamethoxazole (80–400 mg) three times a week after dialysis. The patient has not had recurrence of respiratory complaints since completion of treatment for PCP and is expected to complete a 12-month minimum course of trimethoprim-sulfamethazine prophylaxis in September 2019.

## 3. Discussion

Pneumocystis pneumonia is caused by the human pathogen *Pneumocystis jirovecii*. *Pneumocystis jirovecii* is a spherical or cup-shaped, thick-walled cyst that typically measures 6–8 *μ*m in diameter. This organism, which is classified as a fungus, is believed to be transmitted by inhalation [1]. *P. jirovecii* is commonly encountered early in life and persists in an inactive or latent state due to immunocompetence [1, 2]. The incidence of this disease surged during the acquired immunodeficiency syndrome (AIDS) and HIV epidemic, with a peak number of cases in 1987 [3, 4]. Following the introduction of HAART in the mid-1990s, the frequency of occurrences diminished dramatically, by 80% [2].

The classical presentation is an indolent process characterized by fever, cough, dyspnea, and tachypnea. Physical examination is often nonspecific, and pulmonary auscultation varies from normal to rales. Initial imaging of chest radiograph (CXR) will demonstrate bilateral, diffuse interstitial, and alveolar infiltrates. High-resolution CT has a higher sensitivity than CXR and will show patchy ground-glass opacities, predominating in perihilar region of the lungs. Less commonly, CT detects cyst formation, thick-walled cavitary nodules, and noncavitary nodules [2–4].

PCP is diagnosed by visualization of either the intracystic sporozoite or extracystic trophozoite in respiratory secretions [1]. Respiratory secretions are collected from induced sputum, fiberoptic bronchoscopy with BAL, and transbronchial biopsy. It can also be diagnosed via biopsy by thoracotomy or video-assisted thoracoscopic surgery. *P. jirovecii* does not grow in vitro in fungal media, and the trophic form is identified following the application of specific stains [1]. These stains include Grocott's methenamine silver, cresyl violet, Gram-Weigert, or toluidine blue O stain [1, 3]. Induced-sputum monoclonal antibodies have a higher sensitivity and specificity than conventional stains. Conventional PCR and quantitative PCR methods have been developed; however, evidence regarding these techniques demonstrates limited clinical significance in comparison to other methods of diagnosis [3].

PCP is treated with a 21-day course of trimethoprim-sulfamethoxazole (TMP-SMX) ((TMP) 15–20 and (SMX) 75–100 mg/kg/day), assuming normal renal function. This is typically divided into three or four doses per day. Side effects to monitor include rash, fever, increased serum creatinine, neutropenia, and transaminase elevations [2, 5, 6]. It is important to note that TMP-SMX is the appropriate treatment, despite prophylactic management [6]. In addition, several studies have demonstrated a benefit in mortality if steroids are prescribed alongside antipneumocystis therapy, especially in those with oxygen exchange abnormalities on presentation [7].

Prophylactic management of TMP-SMX is recommended as secondary prophylaxis. In patients infected with HIV, this prophylactic antibiotic regimen is indicated following PCP treatment. It can be discontinued if the patient is on HAART, has an undetectable viral load, and has a CD4 count >200 cells/*μ*L for at least three months. Although other sources recommend considering discontinuation in HIV-infected patients with a CD4 count of 100–200 cells/*μ*L and an undetectable viral load for at least 6 months [2, 5, 8], national CDC and NIH compiled recommendations suggest life-long secondary prophylaxis in the setting of PCP diagnosis with CD4 count greater than 200 cells/*μ*L. However, in select cases, discontinuation can be considered due to limited disease prevention and medication side effects [2]. If there is a recurrence of PCP despite CD4 >200 cells/*μ*L, then lifelong prophylaxis should be strongly considered [1].

Ultimately, this patient's case of PCP was attributed as a consequence of hypersensitivity pneumonitis management and antibiotic prophylaxis failure. However, it is possible the patient's original presentation was due to PCP, but was unable to be diagnosed due to suboptimal bronchoscopy.

## 4. Conclusion

*Pneumocystis jirovecii* pneumonia is a common pathogen causing pneumonia in humans. This case illustrates that PCP should be considered in an HIV-infected patient with undetectable viral count and significant CD4 count (>487 cells/*μ*L), despite limited case reports. Furthermore, PCP prophylaxis does not exclude PCP as a diagnosis, especially in the setting of immunosuppressants such as steroids.
